# Slow touch targeting CT-fibres does not increase prosocial behaviour in economic laboratory tasks

**DOI:** 10.1038/s41598-018-25601-7

**Published:** 2018-05-16

**Authors:** Lisa Anna Rosenberger, Anbjørn Ree, Christoph Eisenegger, Uta Sailer

**Affiliations:** 1 Neuropsychopharmacology and Biopsychology Unit, Department of Basic Psychological Research and Research Methods, Faculty of Psychology, University of Vienna, Vienna, Austria; 2 Faculty of Medicine, Institute of Basic Medical Sciences, Dept. of Behavioural Sciences in Medicine, University of Oslo, Oslo, Norway

## Abstract

Field studies have demonstrated that humans become more generous, helpful and compliant after having been touched by another person. Here, we explored whether these effects are larger for touch activating the C-tactile (CT) fibres, as it is ascribed a particular role in establishing and maintaining bonds and affiliative interactions. The role of CT-targeted and non-targeted touch on prosocial behaviour was investigated in three different experiments using a trust game and a task measuring individual differences in social value orientations (the SVO task). Whereas participants in general acted prosocially, there was no influence of CT-targeted touch on prosocial behaviour, both in comparison to non-CT-targeted control touch and visual (non-tactile) stimulation. The null findings were further corroborated by Bayesian statistics. Thus, under the controlled laboratory conditions employed, CT-targeted touch did not play a particular role in prosocial behaviour. This indicates that touch does not increase prosocial behaviour in the absence of meaningful social and psychological connotations. Any touch related effects on prosocial behaviour likely depends on the ecological validity of the situation.

## Introduction

Touch plays an important role in initiating and maintaining social affiliation and attachment, as has been shown for primates^1–3^ rats^4^, and humans^5–7^. Besides its role for establishing and upholding bonding, touch has also been found to increase helping behaviour and compliance with requests. As a now classic example, restaurant customers who were briefly touched by a waitress gave larger tips^8,9^, and supermarket shoppers were more willing to take part in alleged research interviews following touch^10^. Touch also increased compliance with the request to look after a “large and very excited” dog while the owner went to a shop where dogs were prohibited^11^. Many more field studies^12–14^ and one study using an economic game^15^ showed that touch recipients are more likely to be unselfish and generous (for reviews, see^16,17^).

These studies have in common that they only applied one type of touch and compared it to no use of touch. However, this is not sufficient to determine what kind of touch is most effective (for example, tapping versus stroking), and what the underlying mechanisms may be. Moreover, being touched may induce arousal and other psychological (e.g., assumed affection) and physiological (e.g., heart rate increase) effects which would not be present in a control condition of no touch. Therefore, in order to understand the specific effects of touch, experimental approaches are needed that disentangle these potential factors.

Since most of these existing studies were field studies, there was also a limit to how well the variable touch could be controlled. However, different characteristics of the touch applied may lead to different effects on prosocial behaviour. Humans have both a rapid touch system, where signals are transmitted via myelinated, fast conducting A β afferent nerves, and a slow touch system consisting of unmyelinated C fibres with a conduction velocity about 50 times lower. A particular type of C fibres, the C-tactile (CT) fibres, are present only in the hairy skin of humans^18–20^. They are believed to filter information relevant to hedonic or social aspects of gentle touch, before transmitting it to the brain where it is processed within affective brain networks (for a recent review, see^21^). CT-fibres are maximally activated to stimuli at skin temperature^22^ and by gently stroking the participant at a velocity similar to that of caressing^23,24^. Since they appear to respond to stimuli signalling interpersonal caress, they are thought to represent a peripheral mechanism to promote affiliative behaviour^22,25^. Indeed, the “social touch hypothesis” assumes a relationship between CT-targeted touch and bonding, attachment, and social perceptions^21^. Given these previous findings and theoretical assumptions, it seems plausible to hypothesise that the positive effect of touch on social behaviour is mediated via CT-fibres. The present study set out to test this hypothesis in an experimentally controlled laboratory setting, and to establish the specific role of CT-targeted tactile stimulation in prosocial behaviour. To this end, we conducted three experiments with two different economic tasks and different control conditions under controlled laboratory conditions. Based on the literature review above, we hypothesised that prosocial behaviour would be enhanced following CT-targeted touch.

To determine the influence of CT-targeted touch on prosocial behaviour, we employed a repeated trust game^26^ in two experiments, a classic two-player task of behavioural trust and reciprocity. The participants always played in the role of trustee, where they had to make decisions on how much of the entrusted money they wanted to send back to their interaction partner. Thus, the prosocial measure in experiments 1 and 2 was the back-transfer amount, or in other words the amount of reciprocity displayed to the interaction partner. The other player was either identical to the person providing touch (experiment 1), or not (experiment 2). In experiment 3 we employed a Social Value Orientation task (SVO^27^) which measures prosocial behaviour in terms of concern for other people’s pay-off. Here, the participant makes decisions on how to allocate a monetary amount between themselves and an anonymous other. In all three experiments, the monetary decision of the participant was measured after having either received CT-targeted touch (3 cm/s) or control touch (tapping in experiments 1 and 2, and very slow touch in experiment 3), both executed with a soft brush. A brush was preferred to the more ecologically valid hand, because previous studies have shown reliable CT-fibre activation using a brush^22,23^. Moreover, a brush allows for more controlled tactile stimulation, since it is independent of skin temperature and humidity, and also because the force can be better monitored via the bending of the hairs.

In experiment 3, an additional control condition with a moving visual stimulus was employed in order to investigate whether a potential increase in prosociality was specific to the tactile stimuli, or whether it is just the pleasantness of any sensory stimulus that leads to an effect. In order to provide evidence to support the null hypothesis of no differences between conditions, Bayesian analysis was performed. Instead of computing a p-value, Bayesian analysis computes the odds favoring the null hypothesis or a plausible alternative in light of the data^28^. This allows us to determine the strength of the evidence for CT-targeted touch (not) having an effect on prosociality.

## Results

### Experiment 1

Pleasantness for CT-targeted touch was rated higher than for control touch (F(1, 98) = 6.907, p < 0.001). Intensity was not rated as being different for the two touch types (F(1,98) = 0.053; p = 0.818).

The return ratio was larger with a multiplication factor of 6, that is when the investment was multiplied with 6, than with a multiplication factor of 3 (χ^2^ (1) = 53.553, p < 0.001), but it did not differ for touch types (χ^2^ (1) = 0.098, p = 0.754), or pleasantness (χ^2^ (1) = 0.578, p = 0.447). There was also no interaction between multiplication factor, touch type, or pleasantness (see Supplementary Material Results 1 for details on the results, and Table 1 for means and standard deviations of the ratings and return ratios).

### Experiment 2

As in experiment 1, pleasantness for CT-targeted touch was rated higher than for control touch (F(1, 156) = 29.65; p < 0.0001). Intensity was not rated as being different for the two touch types (F(1, 156) = 0.022; p = 0.882). As in experiment 1, the return ratio was larger with a multiplication factor of 6, than with a multiplication factor of 3 (χ^2^ (1) = 131.020, p < 0.001). Pleasantness was found to influence the return ratio (χ^2^ (1) = 4.602, p < 0.05): higher pleasantness ratings were associated with higher return ratios. The return ratio was not different for the two touch types (χ^2^(1) = 0.441; p = 0.506), and there was also no interaction between multiplication factor, touch type, or pleasantness (see Supplementary Material Results 1 for details on the results, and Table 1 for means and standard deviations of the ratings and return ratios).

### Pooled data from experiment 1 and 2

The data from experiments 1 and 2 were pooled in order to investigate potential differences between the experiments, and to additionally explore the effect of hypothesis guessing.

#### Pleasantness and intensity

**Figure 1:**
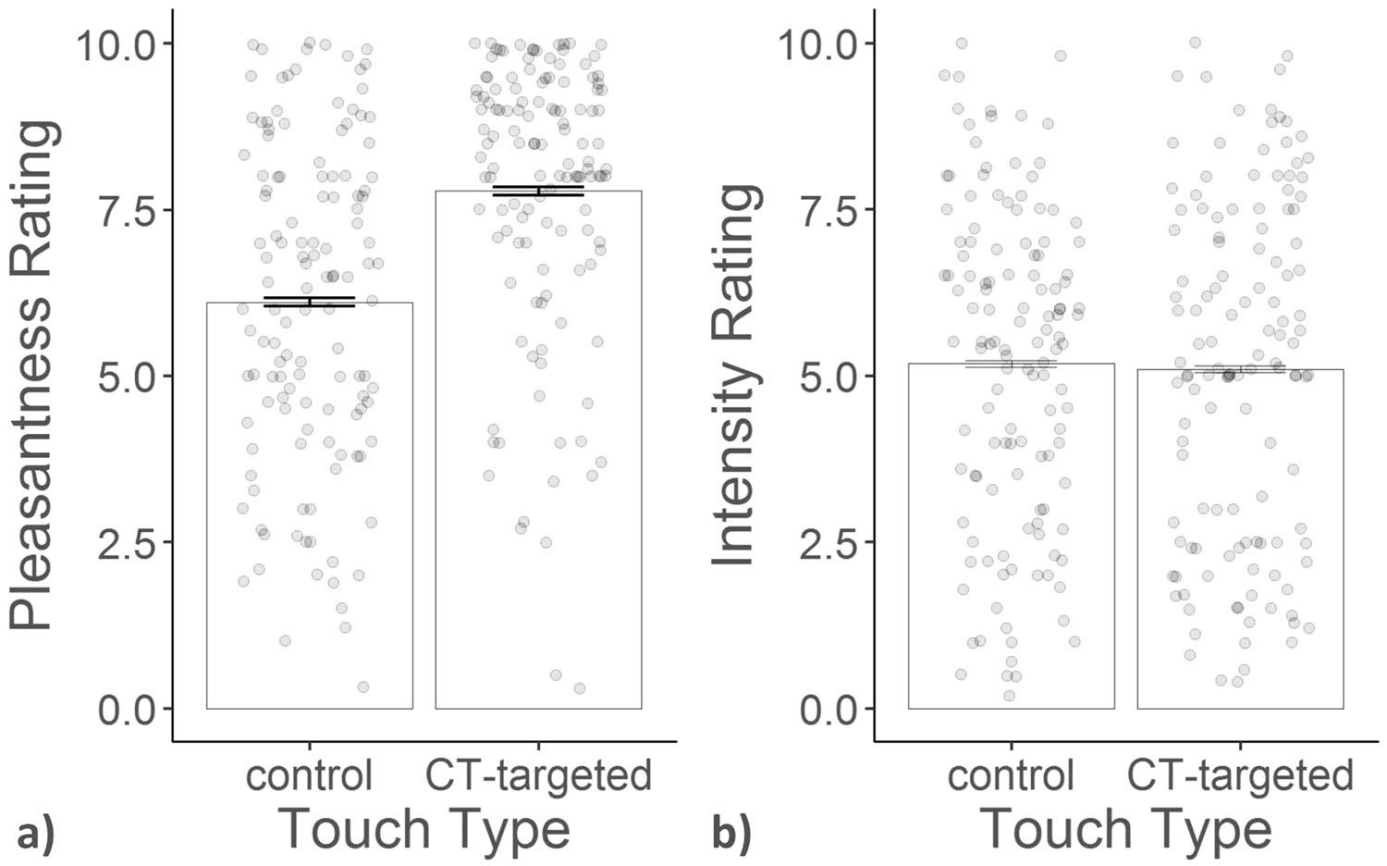
Mean pleasantness (panel a) and intensity (panel b) with within-subject standard error of the mean (SEM) and individual single-trial ratings of experiment 1 and 2 for CT-targeted and control touch.

CT-targeted touch was rated as more pleasant than control touch (see Fig. 1) (F(1, 250) = 34.819, p < 0.0001), with a Bayes factor (BF) of 7.714. This means that the data support a difference in pleasantness ratings between CT-targeted touch and control touch over no difference between the two by almost a factor of 8. At the same time, both touch types were rated as equally intense (F(1, 250) = 0.066, df = 1, p = 0.798), with a BF of 0.107. There were no differences in pleasantness or intensity ratings between experiment 1 and 2, nor was there an effect of hypothesis guessing on the ratings (all F < 2.139).

#### Return ratio

**Figure 2:**
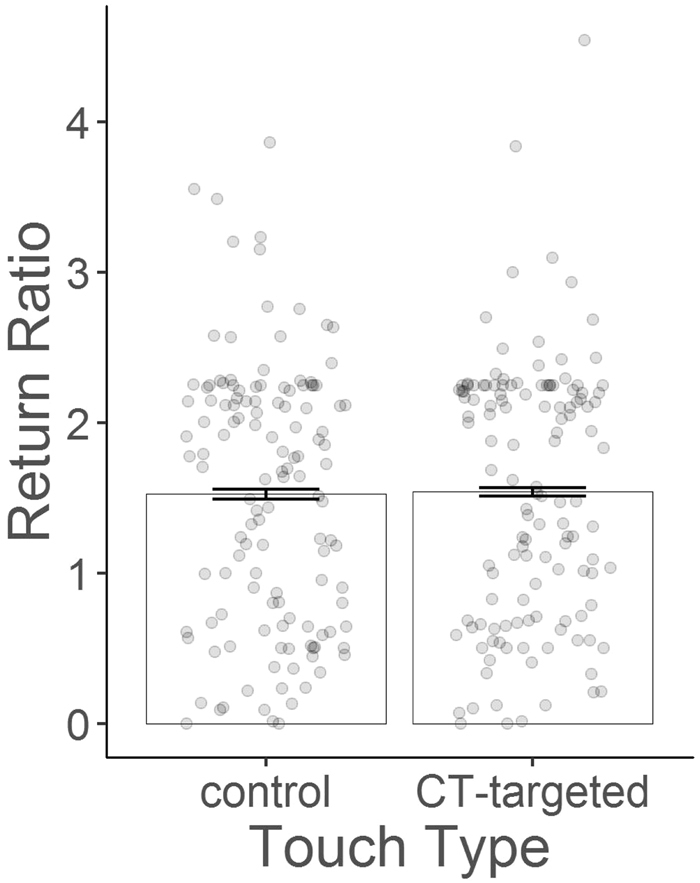
Mean return ratio with SEM and individual data points (averaged for each participant from experiment 1 and 2) for CT-targeted and control touch.

Return ratio following both types of touch was larger than 1 (see Fig. 2), indicating that participants on average returned more than the investor had invested. However, there was no general difference in the return ratio depending on the type of touch (χ^2^(1) = 0.173, p = 0.678, see Supplementary Material Results 2 for a full model description). The Bayes factor analysis revealed a BF of 0.098 for touch type differences, thus, the data support the null hypothesis of no difference between the two touch types with a BF of 10.25. The BF for touch type differences with multiplication factors of 3 and 6 were both 0.136. Again, the data support an absent difference between the two touch types with a BF of 7.34. Furthermore, the BF on the interaction between pleasantness and touch type (thus the probability that a return ratio difference between the touch types depends on pleasantness ratings, given the data) was 0.225. Thus, in contrast to our hypothesis, both the mixed model analysis and the Bayes factor analysis indicate that there was no effect of touch type on return ratios.

**Figure 3:**
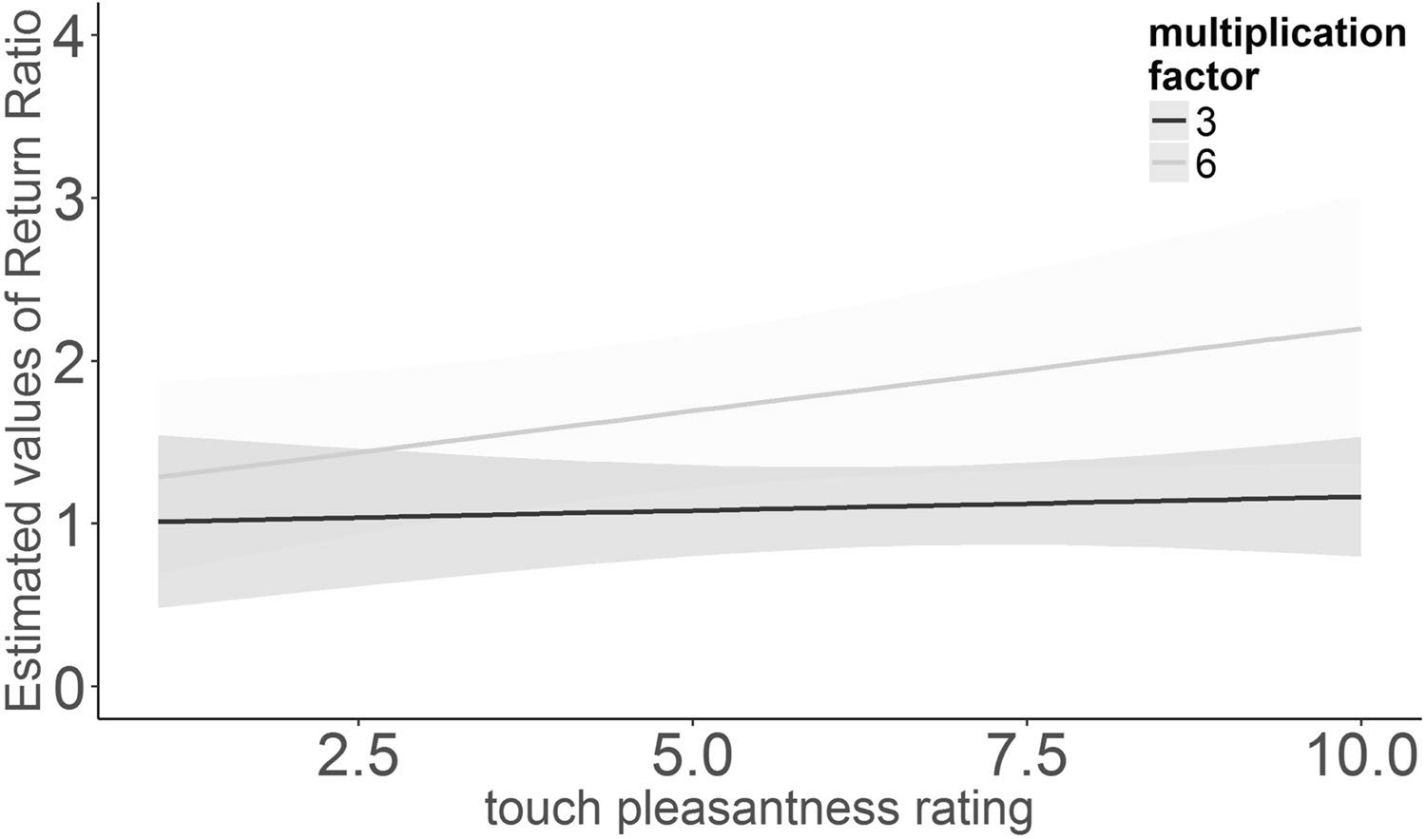
Regression line of estimated return ratios from linear mixed model analysis with 95% confidence intervals (shaded areas) for touch pleasantness ratings (both CT-targeted and control touch) of participants who guessed the study’s hypothesis split by multiplication factor.

As to hypothesis guessing, 6 participants in experiment 1 (12%) and 14 participants (18%) in experiment 2 correctly guessed the hypothesis of our study despite the cover story. Hypothesis guessing affected the return ratio, as shown by a significant interaction between multiplication factor and hypothesis guessing (χ^2^ (1) = 15.789, p < 0.01), as well as a significant interaction between multiplication factor, hypothesis guessing and pleasantness (χ^2^ (1) = 7.226, p < 0.01, see supplementary material Results 2). To further explore this 3-way interaction, an additional analysis was performed separately for participants who guessed and did not guess the hypothesis. For participants who guessed the hypothesis, there was a significant interaction between multiplication factor and pleasantness (χ^2^ (1) = 3.999, p < 0.05), see Fig. 3. In trials with higher multiplication factor, the return ratio increased with increasing pleasantness ratings.

**Figure 4:**
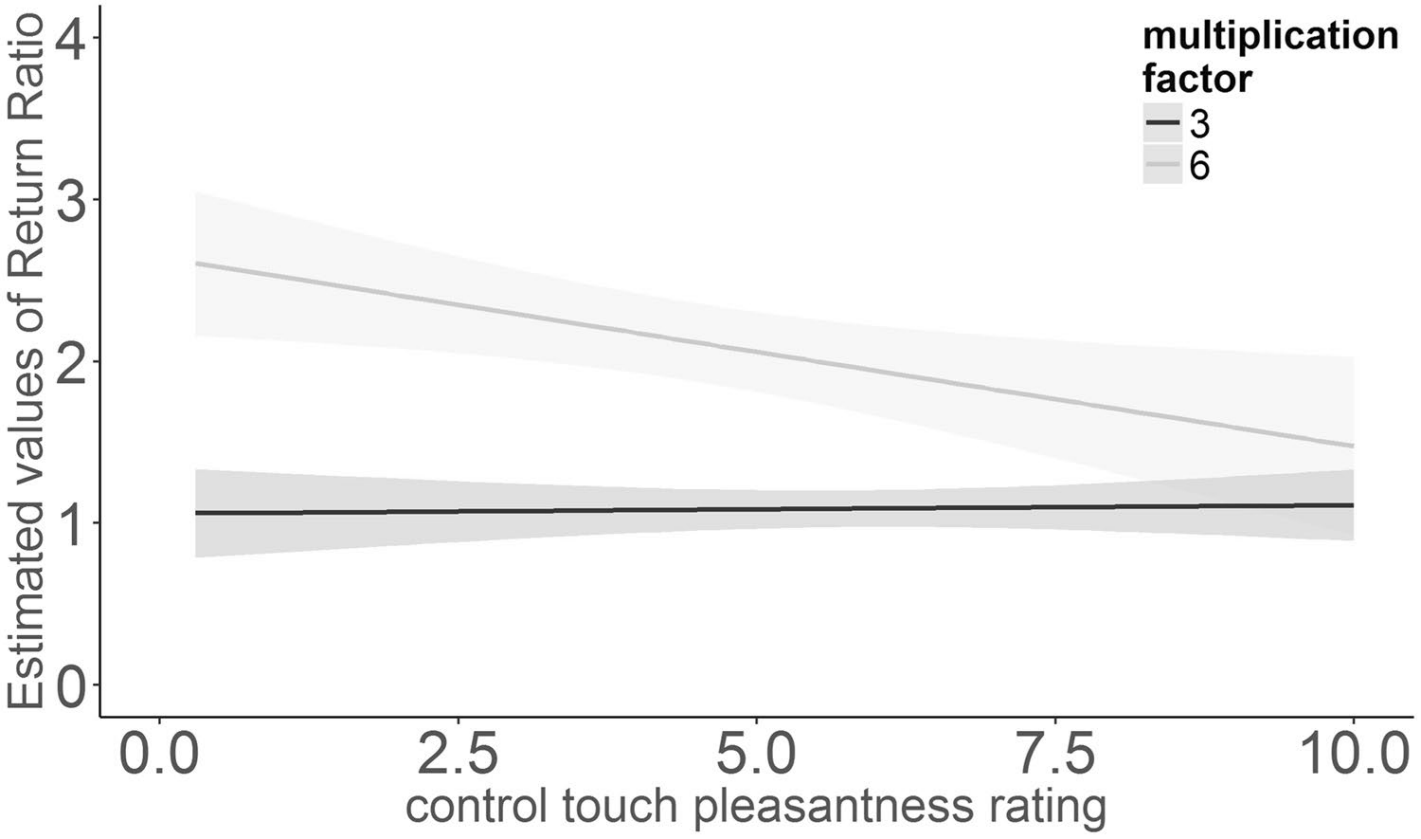
Regression line of estimated return ratios from linear mixed model analysis with 95% confidence intervals (shaded area) for different control touch pleasantness ratings of participants who did not guess the study’s hypothesis split by multiplication factor.

For participants who did not guess the hypothesis, there was a significant interaction between multiplication factor, touch type, and pleasantness (χ^2^ (1) = 4.912, p < 0.05). Calculating separate analyses for both touch types (see Supplementary Material Results 3 for full model description) showed that this interaction was due to an effect of control touch, where higher pleasantness ratings in trials with a high multiplication factor were associated with lower return ratios (interaction between multiplication factor and pleasantness; χ^2^ (1) = 13.234, p < 0.001), see Fig. 4.

Thus, whereas participants who correctly guessed the hypothesis returned more money the more pleasant they evaluated the touch, participants who did not guess the hypothesis returned less money the more pleasant they evaluated the touch. The latter did, however, only apply to control touch and with the higher multiplication factor.

### Experiment 3

**Figure 5:**
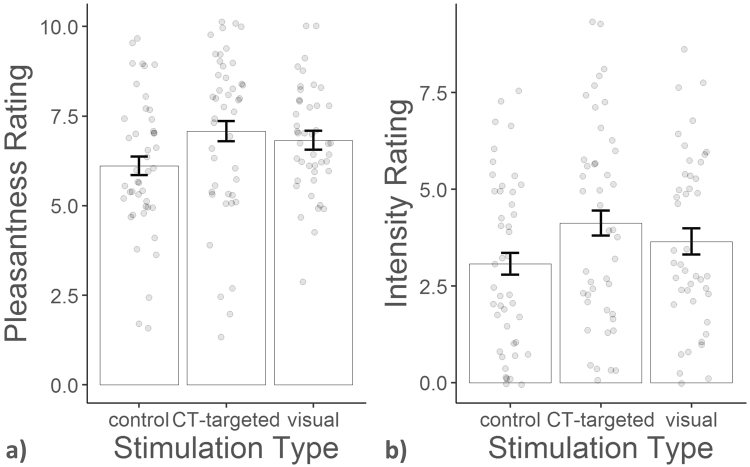
Mean pleasantness (panel a) and intensity (panel b) with within-subject SEM and individual ratings for different sensory stimulation.

As in experiments 1 and 2, pleasantness was rated differently for the three types of stimulation (see Fig. 5, χ^2^ (2) = 6.961, p < 0.05), with CT-targeted touch being evaluated as more pleasant than control touch (t(86) = 2.542, p < 0.05). Bayes factor analysis revealed that a difference between the two touch types is 2.135 more likely than no difference between the two ratings, given our data. However, CT-targeted touch was not rated as more pleasant than visual stimulation (t(86) = 0.661, p = 0.787), which is supported by a BF of 0.257. There was a trend for intensity to be rated differently for the three types of stimulation (χ^2^ (2) = 5.639, p = 0.060), which was driven by tendentially higher intensity ratings for CT-targeted touch than for control touch (t(86) = 2.371; p = 0.052). However, Bayes factor analysis actually does not support the existence of a difference in intensity ratings between the three types of stimulation (BF = 0.242).

**Figure 6:**
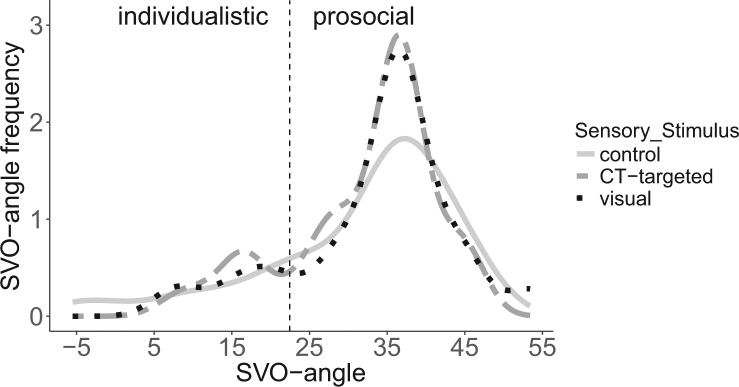
Distribution of SVO angles following different sensory stimulation. Behavioural categorization is based on the following ranges: “altruistic” (>57.15 deg), “individualistic” (between −12.04–22.45 deg), “prosocial” (between 22.45–57.15 deg), or “competitive (<−12.04 deg)”.

Following all three types of stimulation, all of the participants had an SVO angle that classified them as either individualistic or – the majority – as prosocial (see Fig. 6). None of them fell into the competitive or altruistic range. For three participants following control touch and for one participant following visual stimulation, an SVO angle could not be calculated because of inconsistent behaviour.

SVO angles did not change depending on type of stimulation, or its pleasantness. There was only a tendency for the SVO angle to differ between the three types of stimulation (χ^2^ (2) = 5.756, p = 0.056, see supplements Results 4). No other effects or interactions were significant. Post-hoc tests revealed that the SVO angle was higher following visual stimulation (M = 33.519, SD = 10.470) than control touch (M = 31.463, SD = 12.491; t(78.52) = −2.662; p < 0.05). The SVO angle for CT-targeted touch (M = 32.148; SD = 9.491) was in-between visual stimulation and control touch, and did not differ significantly from any of them (all ts < 1.53). The BF for differences in the SVO angle between stimulation types was 0.104. Thus, our data support an absent difference between the stimulation types with a BF of 9.593. The BF for differences in SVO between stimulation types depending on pleasantness (thus the stimulation type x pleasantness interaction) was 0.146, and therefore, gave more support for an absent effect. Thus, as in experiments 1 and 2, and against our hypothesis, CT-targeted touch did not increase prosocial behaviour.

## Discussion

The goal of the study was to test the hypothesis that slow brushing with an optimal velocity to activate CT-fibres increases prosocial behavior. To test the hypothesised prosociality-enhancing effect of touch, three experiments with two different behavioural economic laboratory tasks and different control conditions were performed. However, following CT-targeted touch (and compared to control touch or visual stimulation) neither reciprocity to the person who touched the participant, nor reciprocity to an unrelated person in the trust game were altered. Additionally, prosocial dispositions as measured with the SVO also did not shift.

In general, our data replicated some typical findings from both the Repeated Trust Game and the SVO. For example, it is well-established that participants are willing to return positive amounts^29,30^. It has also been shown previously that the same participants return a larger amount if the investment has been multiplied by a larger factor compared to a smaller factor^31–33^ (however, see^29^ for opposite results in between-subjects designs). We were also able to replicate the effects from the Repeated Trust Game during a second experiment, where the change in some experimental details did not affect the participants’ behaviour. Importantly, experiment 2 showed that the absent effect of CT-targeted stimulation on trust game behaviour in experiment 1 was not due to playing with the experimenter, or to conducting the task in an Excel-sheet.

Based on their SVO-angle, the majority of participants were classified as prosocial, and the remainder as individualistic. That is, prosocial participants chose to allocate the money in a manner that evenly distributed the money between “self” and “other”, as opposed to individualistic participants who opted to allocate a larger portion of the allocated money to themselves. This is in accordance with findings from the original study^27^, where 59% of participants were prosocial, 35% were individualist and the remainder competitive. Thus, our participants seem to behave similarly to other healthy participants in studies with the same tasks.

Although we were able to replicate basic findings of both tasks reported in literature, and from the Repeated Trust Game in two experiments with different samples, our data did not support our main hypothesis regarding CT-targeted touch. Many field studies in contexts as different as stores, restaurants, the street and the classroom demonstrated that casual touch leads to more favourable attitudes and behaviour towards the toucher, as well as to larger compliance and increased helping behaviour (for reviews, see^16,17^). Our data did not provide evidence for increased prosocial behaviour following CT-targeted touch, or following touch compared to visual stimulation. This was despite the fact that CT-targeted touch was consistently experienced as more pleasant than control touch. Such an absent influence of different touch types on behaviour, despite differences in their experienced pleasantness, is in line with findings that gentle touch in general, irrespective of CT-optimality, elicits affiliative emotions^34^.

CT-fibres are known to show depressed responses with repeated stimulation, a phenomenon called fatigue. However, human CT’s continue to respond to repeated touch^18,20^ as opposed to non-human animals, whose responses quickly depress with repeated mechanical stimulation^35^. When stimulated every 3 seconds, CT responses in humans became shorter, but maintained the initial peak firing frequency^18^. This may suggest that the extended brushing period on the same area on the arm in experiment 1 and 2 led to decreased CT responding. However, in experiment 3, stroking was alternated between 2 different areas on the arm, which did not lead to different pleasantness responses. Moreover, each brush stroke can be expected to activate a large number of CTs. Therefore, it appears unlikely that the absent effect of CT-targeted touch on prosocial behaviour is due to fatigue of CT-fibres. Nevertheless, as little is known about fatigue in CTs and its effect on subjective experience, further studies are warranted.

Regarding the comparison of tactile versus visual stimulation, prosocial behaviour was not different for visual stimulation either. Interestingly, participants showed opposing effects of touch on prosocial behaviour during the Repeated Trust Game, as measured with the return ratio, depending on whether they guessed the hypothesis correctly or not. This highlights the general importance of assessing the presence of hypothesis guessing when using the trust game. It needs to be taken into consideration, however, that the specific sample in experiment 1 and 3 may have increased the tendency and particularly the accuracy to guess the hypothesis, as it contained a number of psychology and medical students. Both can be expected to know about the use of deception in experiments and/or have some knowledge about the tactile system. However, in experiment 2 only a minority of the participants were students, but still the results were similar to the ones from experiment 1.

Besides showing no advantage of CT-targeted touch compared to control touch in eliciting prosocial behaviour, the findings of experiment 3 also documented a lack of enhancement of prosocial behaviour following touch compared to a visual control condition. This was a rather surprising finding. One reason for this absent difference in prosocial behaviour might lie in the experienced pleasantness of the two stimulations which were rated as similarly pleasant. However, comprehensive analyses showed that pleasantness did not influence the SVO angle. It therefore seems that it was not the similarity in pleasantness for CT-targeted touch and visual stimulation that underlay the absent difference in prosocial behaviour. Similarly, arousal cannot serve to interpret the results, since different arousal following the different types of stimulation would be expected to alter intensity ratings, which was not the case. What could then be the reason for the failure to replicate the field findings of increased prosocial behaviour following touch in our study? It could be speculated that an individual receiving touch may, consciously or subconsciously, interpret the meaning of this behavior^36^. Accordingly, being touched by a waitress or a person asking for a favour in a natural context may be interpreted as a sign of affection^37^ or need^38^. Receiving touch may also indicate physical and emotional closeness to the toucher, social inclusion, and/or felt security^36^. This may in turn lead to positive feelings and beneficial physiological changes. It appears likely that the effect of touch on prosocial behaviour is dependent on one or several of these mediators that we were unable to replicate in a laboratory setting. Moreover, it is well-known that C-fibres are subject to modulation from higher-order brain regions via descending influences (e.g.^39^). Therefore, affective processing of CT-targeted touch is also influenced by top-down cognitive modulation (as reviewed in^40^). For example, the assumed sex of the touch provider^41,42^, concomitant visual^43^ and olfactory^44^ stimuli have all been found to influence the response to soft touch. Thus, early sensory processing will be altered by contextual mechanisms related to aspects such as expectations or the interpretation of touch.

To our knowledge, previous studies investigating the effect of touch on prosocial behaviour in economic games are scarce^15,45,46^. One recent study compared the effect of vibratory touch, an auditory cue or silence on behaviour in the Ultimatum game^45^. In this game, one player offers a portion of an endowment to another player who can either accept the offer or reject it. If the second player accepts the offer, the money is divided according to the proposed division, otherwise, both players leave with nothing. The authors found higher acceptance rates of offers following vibratory touch than silence, but no difference in acceptance rates following touch compared to sound^45^. In a different study on emotional face processing^47^, the authors concluded that auditory and tactile primes rather seemed to affect stimulus evaluation in general by facilitating cognitive processing instead of having differential effects. Such a general facilitating effect may also exist for visual and tactile stimuli in the present study.

In a different study, massage increased reciprocity in a one-shot trust game^15^, with participants returning more than twice as much following massage than following rest. However, in that study the difference between the two conditions may have been maximized because of the length and high intensity of the massage on the one hand, and because of the low arousal and possibly boredom in the rest condition on the other hand. Nevertheless, in comparing touch to “nothing”, the design of this study is similar to the many field studies on touch effects on prosociality, which may explain why similar results were found. The current control condition with visual stimulation necessitated task engagement, because participants were required to rate the videos and therefore, to attentively watch them. This raises the possibility that task engagement, or the lack thereof, may have an influence on prosociality. It has been shown previously that boredom can affect decision-making in terms of choosing a simpler strategy^48^. A potential effect of boredom on prosocial behaviour could be an interesting question to investigate in further studies.

To conclude, it has been suggested that CT-fibres constitute a specific pathway for signaling touch with social-affective meaning^25,49^. Our data suggest that touch that particularly activates this pathway does not influence social behaviour in the present laboratory context. We reason that in order to obtain such an effect, the social context may be crucial. Potentially, touch needs to be understood as expressing affection, not as a rather neutral stimulation as part of an experiment. Therefore, future studies on similar research questions should increase the potential affiliative meaning even in the laboratory context.

## Methods

### Participants

Only participants who had not previously participated in other touch or economics related experiments were eligible for the studies. This was assessed when appointments were booked. For experiment 1, 50 healthy participants, 29 women and 21 men, were recruited via announcements on boards at the University of Gothenburg, Sweden. They were aged between 19 and 72 years, with a mean age of 30 years (SD = 12), and the majority of them were students. For experiment 2, 79 participants, 56 women and 23 men, were recruited via announcements on university boards and on social media. They were aged between 19 and 76 years, with a mean age of 41 years (SD = 19). 8 of them were medical students and 2 were psychology students. Following experiments 1 and 2, participants were asked what they thought the aim of the study was. The response was registered and coded as hypothesis guessing yes/no to be used in later analyses. Subsequently, participants were fully debriefed about the real aim and all deceptive elements in the experiments. All participants received financial compensation for participation which included a show-up-fee of 200 SEK (~20 EUR) and an additional amount that depended on their actual choices during the Repeated Trust Game (see detailed description below).

For experiment 3, 45 healthy participants were recruited by announcements in various academic institutions in Oslo, Norway. The theme of the announcements focused on how various forms of sensory stimuli could potentially affect decision making, empathy and perceptions of various phenomena. Most participants (approximately 75%) were physiotherapy, osteopathy, psychology or medical students. One participant was subsequently excluded due to an acute bout of rheumatoid arthritic joint pain reported in the written consent form. Thus, a total of 27 women and 17 men, aged between 18–47 (M = 25 years, SD = 6) completed the study. Participants were informed that both their own and a future participant’s compensation for participating in the study could be affected by how they chose to allocate the money. However, during the debriefing, the participants were informed that the deception of “self” and “other” was included to ensure full commitment to the procedure of distributing money and all participants received a voucher with a value of 200 NOK (~20 EUR).

All participants provided informed consent before participating in the study. Experiments 1 and 2 were approved by the regional ethical board at the University of Gothenburg, Sweden (Dnr288–15), and experiment 3 by the regional committee for medical and health research ethics South East, Norway (2011/1337), and all were carried out according to the Declaration of Helsinki.

### Procedure

#### Experiment 1

After having signed the informed consent, participants were asked to fill in a form about demographics, potential skin diseases and medication use. The task procedure was similar in experiment 1 and 2 and consisted of the following blocks: tactile stimulation 1, Repeated Trust Game 1, filler task, tactile stimulation 2, and Repeated Trust Game 2. The order of tactile stimulation type (CT-targeted touch and control touch) was counterbalanced between participants. Participants were told that the aim of the experiment was to investigate the effect of different sensory experiences, among which tactile stimulation and hunger, on gaming behaviour. To increase the credibility of the cover story, they were also told not to eat for at least 2 hours prior to the experiment, and later answered two items about their hunger feelings. During the experiment, participants were seated at a table with a PC where all the tasks were administered. The experimenter sat next to the participant at the same table during the tactile stimulations and the repeated trust games. At the end of the experiment, participants were asked what they thought the aim of the experiment was, and if they had noticed anything particular, and were subsequently debriefed and paid.

### Tactile stimulation

Participants were asked to put their left forearm on a vacuum pillow which lay on a table and was adjusted to their arm. A distance of 10 cm was marked with a pen in the middle of the dorsal surface on their forearm. They were asked to put on occluding glasses to block view of their arm, to look straight ahead and to concentrate on the upcoming tactile stimulation. Using a hand-held soft watercolour brush that was 75 mm wide, the experimenter then delivered one of two different tactile stimulations. In the experimental condition “CT-targeted touch”, repeated brushing-strokes were performed in the proximal-distal direction for 3 minutes with a 0.5 s break in-between strokes, i.e. 32 stroking trials altogether. Brushing was performed with a velocity of 3 cm/sec and an approximate force of 0.4N. Brushing with these parameters has previously been found to maximally activate CT-fibres^22,23^. The experimenter was trained in delivering this type of stimulation by performing the brush strokes on a scale and under the guidance of a bar on a computer monitor moving at 3 cm/s to maintain constant pressure and velocity. During the experiment, the experimenter was also guided by the same bar shown on a monitor not visible to the participant.

In the control condition “control touch”, the experimenter had the brush “jump” across the marked area in four equally large steps, i.e. giving the impression that the arm was being prodded at four different positions within a 10 cm distance. The four hopping steps took as much time as one brushing stroke and were performed with a similar force and in the same direction as the brushing, and on the same area of the arm. This stimulation was also performed for 3 minutes. This kind of stimulation was chosen because it was assumed to activate CT-fibres to a lesser extent than slow stroking, while at the same time providing an experience that qualitatively was considerably distinct from stroking in order to increase the probability of an effect. It has previously been shown that this kind of tactile stimulation is perceived as less pleasant than stroking with CT-targeted parameters^50^.

Whereas the majority of field studies on prosocial touch effects used short stimulation, a longer stimulation period of 3 minutes was chosen in order to increase the reliability of ratings, and because previous studies of our group showed that pleasantness and physiological effects of CT-targeted touch change very slowly with repeated stimulation^51–53^. 3 minutes is also an interval in-between the short tactile interventions in field studies and the 15 minute massage in a different laboratory study^15^.

Following each type of stimulation, participants were asked to grade the pleasantness and intensity of the stimulation on a visual analogue scale (VAS) on a paper. Above the VAS for pleasantness, the question “How did you experience the touch?” was written, and the endpoints were “unpleasant” (scored as −10) and “pleasant” (+10). Above the VAS for intensity, the question “How intensive did you experience the touch?” was written, and the endpoints were “weak” (−10) and “strong” (+10).

### Repeated Trust Game

The trust game^26^ is a frequently used and replicated measure of behavioural trust and reciprocity involving the sequential exchange of a monetary endowment^29^. It is played by two players, an investor and a trustee. The investor, who is the first mover, receives an endowment and can transfer a variable amount from his/ her endowment to the trustee. This investment gets multiplied by a specific factor, called a multiplication factor, by the experimenter. For example if the multiplication factor is 2, the investment gets doubled by the experimenter. The trustee receives this multiplied investment and can make a back-transfer to the investor based on the multiplied investment^26^. The amount passed by the investor is assumed to capture trust, and the amount returned by the trustee is assumed to reflect reciprocity^26^.

Participants played this game with the experimenter, i.e. the person touching them. Participants always played in the role of trustee, but were led to believe that this was assigned randomly with a lottery drawing. The endowment size was set to 100 SEK (~11 EUR) and the multiplication factor was either 3 (round 1–5) or 6 (round 6–10). Participants played with the same investor for 10 rounds. Investments were fixed to 70, 90, 80, 70, and 80 SEK for rounds 1–5, and were repeated in rounds 6–10. Participants were instructed that the investor would not see the size of the back-transfer in each round, but would only see the final result of all trials at the end of the experiment. This was done so that participants did not believe their choice would influence the investor’s allocation in the next trial.

Participants were also informed that 10% of the amount they had decided to keep in two randomly selected rounds would be added to their show-up-fee. In fact, these two rounds were not randomly selected, but were the fifth round from block 1 and the last round from block 2. They were also told that the investor would receive the amount transferred back by the trustee from these randomly selected rounds, so that the actual payoff for both players depended on the trustee’s behaviour in the game.

**Figure 7:**
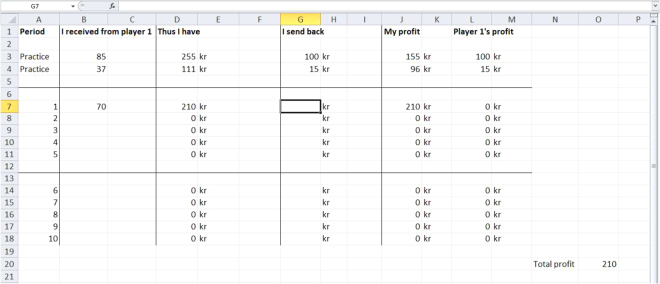
Implementation of the Repeated Trust Game in Experiment 1 (originally in Swedish). Participants always saw the complete Excel sheet. In a given round, the participant received a paper with the investment decision from the experimenter and filled this amount in the column “I received from player 1”. The multiplied amount was automatically displayed in the “Thus I have” column. Then the participant filled in his back-transfer decision in the “I send back” column, which led to the automatic calculation and display of the players profits in the last two columns. The Excel sheet was only visible to the participant throughout the task.

The game was implemented in an Excel sheet (see Fig. 7). Prior to the actual experiment, participants played 2 practice rounds (analogous to the “real rounds” during the experiment) in order to get familiar with the procedure.

### Filler task

Participants were asked to repeatedly estimate the length of a 1 second interval and were given a sweet or salty snack, depending on the participant’s preferences, during 3 breaks. Subsequently, participants again answered a question on paper about how hungry they felt. The snack as well as this question was given in order to support the cover story. Another aim of this filler task was to distract the participants in order to avoid carry-over effects from the first stimulation. The break in between the two conditions was on average 31 min (SD = 6).

#### Experiment 2

Experiment 2 served to replicate the results from experiment 1 with a larger sample and revised procedure. To increase differences between the two stimulation types, a watercolour brush was used to deliver CT-targeted touch as in experiment 1, but the control touch was delivered using a cooled-down steel brush. This was done in order to minimise CT-fibre responding, since they are reported to respond preferentially to tactile stimuli at skin temperature^22^.

**Figure 8:**
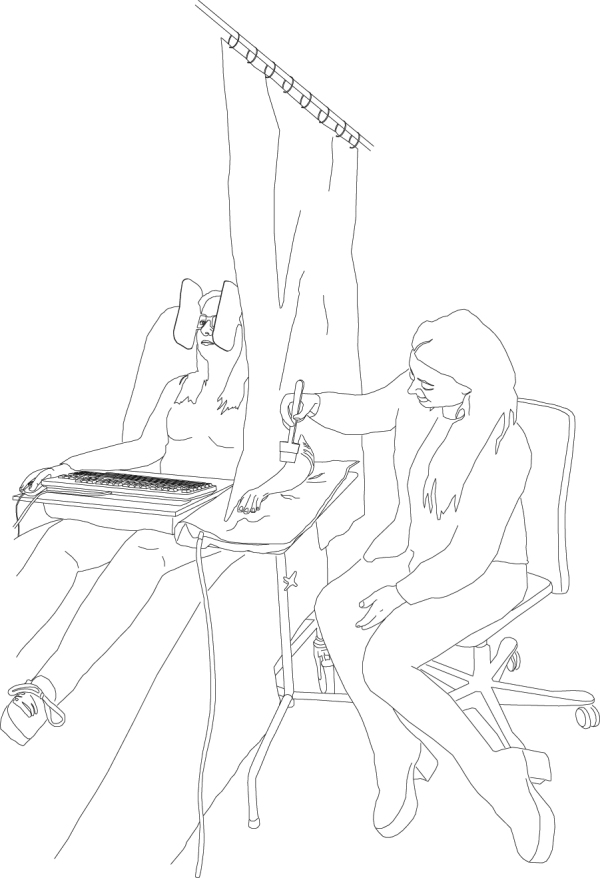
Setup of experiment 2. The participant faced a monitor. The experimenter was seated beside the participant and stroked the participant’s left dorsal forearm with a paintbrush or tapped it with a steel brush.

In experiment 2, participants sat on a hospital bed with a keyboard on their lap and a screen at the height of their eyes in front of them, see Fig. 8. The experimenter was sat to the left of the participants with a dividing curtain in-between, so that participants were unable to see the experimenter/ the other alleged player at all times. In addition, the participant wore occluding glasses that prevented peripheral vision.

In order to reduce a potential influence of the experimenter’s impression on the participants’ behaviour in the Repeated Trust Game, the participants were instructed that they would play the Repeated Trust Game with a colleague of the experimenter, a person they would neither see nor talk to during the experiment. The participants’ view of the experimenter and their own arm was obscured by a dividing curtain. Unbeknown to the participant, the experimenter played the investor. The experimenter opened and closed doors, walked and breathed distinctly in the role of the investor, to increase believability of the deception. Prior to post-experimental de-briefing, the participants were asked about their impression of the investor. None of them mentioned any suspicions about the investor being the same person as the experimenter.

**Figure 9:**
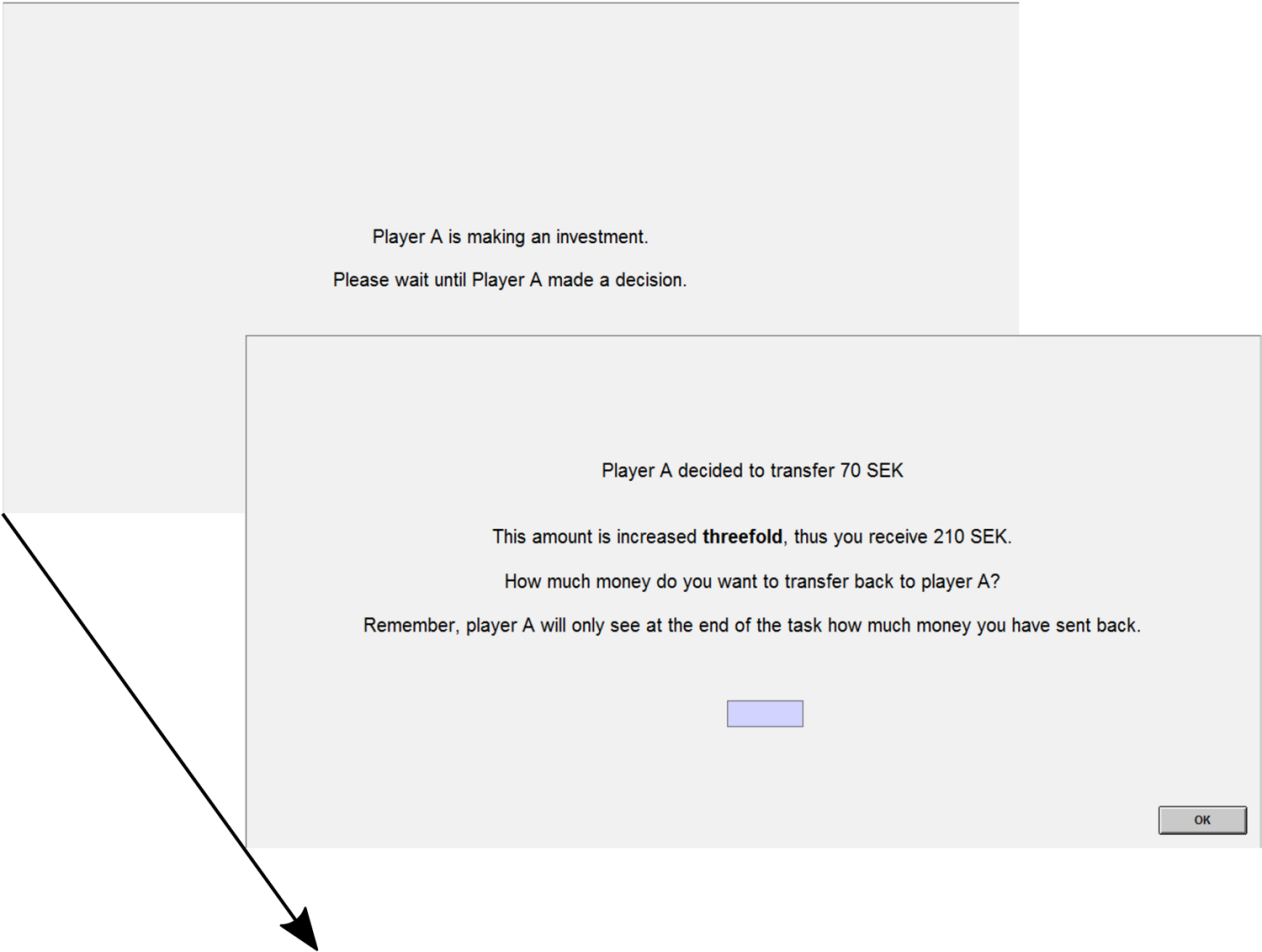
Layout of a trial of the trust game implemented in z-tree (originally in Swedish). The investment screen was displayed between 4–6 seconds. Participants had unlimited time for their back-transfer decision.

The design of the repeated trust game was exactly the same as in experiment 1, except that the task was now implemented in the software z-tree^54^ instead of in an excel sheet (see Fig. 9). Instead of entering back-transfer decisions in an excel sheet, participants now clicked through an interactive task. We opted for a different task interface for the participants to ensure that the results of experiment 1 were not due to the rather bare, mathematical layout of the excel sheet, and also because a standardised and commonly used way of presentation was preferred (see f.e.^55–57^). Again, participants always played in the role of trustee, but were led to believe that this was assigned randomly by z-tree.

#### Experiment 3

Participants were informed that the main purpose of the experiment was to investigate if and how various forms of sensory stimuli influence our values and perceptions of different phenomena. They were further informed that they would be asked to rate the pleasantness and intensity of the sensory stimuli before performing a task that involved allocating money between themselves and a different player. Following signature of the written consent form, electrodes for facial EMG and skin conductance recordings were attached to the participant (results not reported here). Participants were then seated in front of a computer screen with the left arm placed on an inflatable cushion that was adjusted to their arm. A custom-made cap which limited lateral view was provided in order to ensure that the participants did not see their left arm or the experimenter during the experiment. The participants were also equipped with a set of headphones throughout the experiment (AKG, K 271 MkII). The task procedure for experiment 3 consisted of the following blocks: sensory stimulation 1, Social Value Orientation scale 1 (SVO^27^), sensory stimulation 2, SVO 2, sensory stimulation 3, SVO 3.

To further mask the main objective of the study, the participants were also exposed to auditory stimulation with 2 minutes of pink noise which was not followed by an economic task but by a questionnaire pertaining to ratings of facial attractiveness, questions of soft drink preferences and how one would spend an imaginary lottery win. The questionnaire served to further mask the main objective of the study and was therefore not analysed. The order of sensory stimulation types (CT-targeted, control touch, visual and auditory stimulation) was counterbalanced between participants. Visual stimulation was presented as an alternative to a passive “no touch” control condition. A moving, non-tactile control stimulus was chosen, that that was not expected to increase prosociality.

CT-targeted touch was performed as in experiments 1 and 2, however, to control for the possibility of CT-fibre fatigue, the stroking alternated between two equally large areas of the dorsal forearm. Additionally, control touch consisted here of slow brushing at 0.3 cm/s to complement the findings from the previous two experiments which used 30 cm/sec as control touch. The duration of CT-targeted and control touch was 2 minutes each to ensure equal contact time between the conditions. For visual stimulation, participants watched a two minute long film which was edited from six films (Shutterstock Inc; New York, USA) displaying colourful geometrical objects moving at various speeds.

Immediately after the tactile or visual stimulation, the participants rated the pleasantness and intensity of the sensory stimulus using two subsequently appearing visual analogue scales (VAS) as in experiments 1 and 2 on the screen in front of them.

### Social Value Orientation Task

Participants performed the online version of the Social Value Orientation task (SVO^27,58^). The SVO is designed to measure prosocial behaviour, i.e., the concern for other people’s pay-off, with social value orientation corresponding “to the quantity of how much a decision maker is willing to sacrifice in order to make another decision maker better off (or perhaps worse off)^27^.” (p.772). Participants are categorized into one of four different types, based on an angle score calculated from their decisions during the task. A larger SVO angle indicates that participants more often chose the option that maximised the allocation to the other person. On the other hand, a small SVO angle indicates that the participant opted for a larger portion of the allocated money themselves. The angle ranges for the four categories are as follows: “altruistic” (>57.15 deg), “individualistic” (between −12.04–22.45 deg), “prosocial” (between 22.45–57.15 deg), or “competitive (<−12.04 deg)”.

**Figure 10:**
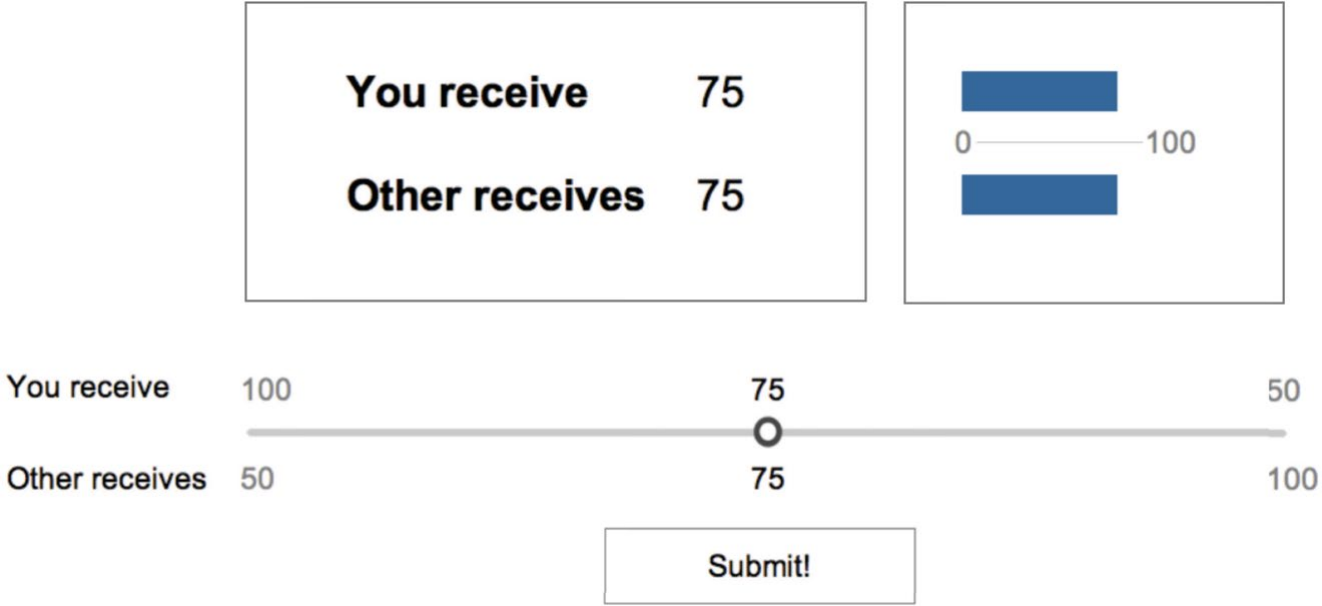
Example trial involving the distribution of NOK 150. The participants were instructed that “you receive” and “other receives” referred to their own and a future randomized participant’s payment. Picture reproduced from^27^ under Creative Commons Attribution 3.0 License.

In the present SVO-task, participants were instructed to allocate between NOK 100 and NOK 150 between themselves and a future random participant. They were told that there were no right or wrong answers, but that they had to carefully consider how they chose to allocate the money as the allocations could affect their own payment and that of the future randomized participant. The participants played the short version, whereby six slots of monetary allocations constitute one game (see Fig. 10). Angle calculations from the website were verified by manual computations by the experimenter. Participants practiced 2–4 rounds prior to the actual experiment.

### Data analysis

All data were analysed with linear mixed models, which are ideally suited for repeated measures designs. There is no need for data aggregation, which increases power and makes it possible to model individual response styles, while at the same time controlling for within-subject dependences of observations^59^. We modelled participants as random intercept and the highest order within-subject interaction as random slope (as recommended by^59^) with the lme4 package^60^ in R. For the reported interactions we calculated Type 3 Sum of Squares and used orthogonal contrasts. Reported main effects are based on Type 2 Sum of Squares. P-values are based on Wald-Chi-square tests from the car package^61^. Post-hoc Tukey tests for factors were computed with the lsmeans package^62^. Results were supplemented with Bayes Factors, which were computed with the BayesFactor package^63^. Plots were created with the packages ggplot2^64^ and sjPlot^65^ in R.

Dependent variables were pleasantness and intensity, and for the Repeated Trust Game the return ratio ($\frac{{back}-{transfer}}{{investment}}$). The return ratio was preferred to the absolute amount returned as a measure of prosocial behaviour because the size of the return ratio is not dependent on the amount invested.

For experiment 1, pleasantness and intensity were compared for the two touch types (CT-targeted, control) in two separate analyses. The return ratio was analysed using a model with the predictors multiplication factor (3, 6), touch type (CT-targeted, control), and pleasantness. An analogous analysis was done for experiment 2. Next, the data of both experiments were collapsed and analysed together. This was done, first, to evaluate whether there were any differences between the experiments, and second, to analyse the effect of hypothesis guessing in both experiments. Participants may correctly guess the intent of the study and consciously, or subconsciously, alter their behaviour. To investigate whether this was the case, hypothesis guessing was added as a predictor to this analysis. Pleasantness and intensity were separately analysed, and the return ratio was analysed with the predictors multiplication factor (3, 6), touch type (CT-targeted, control), experiment number (1, 2), hypothesis guessing (yes, no), and pleasantness.

The central question of experiment 1 and 2 was to determine whether the return ratio is larger after CT-targeted than control touch (hypothesis H1). In contrast, the null-hypothesis (H0) would assume no difference between the two types of touch. Conventional frequentist inference statistics only allows testing H1-hypotheses by rejecting the null-hypothesis. However, if the null-hypothesis cannot be rejected, it does not automatically mean that it is true. Bayesian statistics^66,67^ allow to determine whether the data provide stronger evidence for H1 or the null-hypothesis. The Bayes factor B means that the data are B times more likely under the H1 than under the null-hypothesis. If the Bayes factor (BF) is larger than 1, support for the H1 is stronger, whereas a BF smaller than 1 means that support for the H0 is stronger, given the data. Conventionally, a BF > 3 can be interpreted as substantial evidence, whereas a BF > 10 is considered strong evidence for the model under consideration^68^. However, these thresholds should be used cautiously as rules of thumbs (see^69,70^ for an overview of BF interpretation), which is why we refrain from referring to these thresholds in the results section. BF were calculated for pleasantness and return ratio for the factor touch type, and for return ratio also on the interaction between pleasantness and touch type. BF were calculated for the pooled data only for variables where the mixed model found no difference between the experiments. We used the default prior setting of the BayesFactor package, which is described in detail in^71^ for regression models and in^72^ for ANOVA designs. The default settings specify priors for the variance, the intercept (in the regression models), and the mean of standardized effect sizes of the null and the alternative hypotheses. For the null hypothesis the prior for the mean is 0, and the variance is a non-informative Jeffreys prior. For the alternative hypothesis the priors for the variance and the intercept are also non-informative Jeffreys priors, for the mean it is a multivariate Cauchy distribution with a scale value of 0.5.

For experiment 3, the dependent variables were pleasantness, intensity, and the SVO angle. Pleasantness and intensity were separately analysed for the 3 stimulus types (CT-targeted, control, visual). The SVO angle was analysed using a model with the predictors stimulus type (CT-targeted, control, visual) and pleasantness. BF were calculated for SVO angle and pleasantness.

### Data availability statement

Data and analyses scripts are available at the open science foundation website^73^.

## Electronic supplementary material

Supplementary material
