# Supplementary material for: Slow touch targeting CT-fibres does not increase prosocial behaviour in economic laboratory tasks
Source: Sci Rep. 2018 May 16;8:7700. doi: 10.1038/s41598-018-25601-7 (PMC5955966; doi:10.1038/s41598-018-25601-7)
Supplement: Supplementary file 1 — Supplementary material [file 41598_2018_25601_MOESM1_ESM.pdf]

Supplementary materials

Slow touch targeting CT-fibres does not increase prosocial behaviour in economic laboratory tasks

Lisa Anna Rosenberger, Anbjørn Ree, Christoph Eisenegger, Uta Sailer

In all tables the following information about the random effects of the linear mixed models are added: ICC = intra-class-correlation, N = amount of random intercepts, σ^2 = random within group variance, τ_{00} = random between- group variance, ρ_{01} = random-slope-intercept correlation, R^2 = difference of the total variance of the null and full model divided by the total variance of the null model. This information is derived from the "sjPlot" package in R (Lüdecke, 2017). All reported main effects are computed with type 2 sum of squares and all interactions with type 3 sum of squares.

Results 1

Table 1. *Means (m) and standard deviations (SD) of the ratings and the return ratios of experiment 1 and 2.*

	CT-targeted touch		control touch	
	m	SD	m	SD
pleasantness rating				
experiment 1	7.78	2.07	6.63	2.30
experiment 2	7.79	2.15	5,78	2.48
intensity rating				
experiment 1	5.36	2.52	5.47	2.30
experiment 2	4.94	2.64	5.00	2.50
return ratios				
experiment 1	1.37	1.23	1.29	1.24
experiment 2	1.65	1.01	1.67	1.12

Table 2. *Linear mixed model results of return ratio in experiment 1 predicted by multiplication factor, touch type, touch pleasantness rating and their interactions.*

	Return Ratio - experiment 1				
	χ^2	df	p	std. Beta	95% CI
**Fixed Part**					
Multiplication Factor	53.553	1	< 0.001	-0.31	-0.50 - -0.12
Touch Type	0.098	1	0.754	-0.05	-0.28 - 0.19
Pleasantness Rating	0.578	1	0.447	0.04	-0.04 - 0.11
Multiplication Factor x Touch Type	0.378	1	0.539	0.05	-0.12 - 0.23
Multiplication Factor x Pleasantness Rating	0.001	1	0.973	-0.00	-0.18 - 0.18
Touch Type x Pleasantness Rating	0.360	1	0.549	0.07	-0.16 - 0.31
Multiplication Factor x Touch Type x Pleasantness Rating	1.046	1	0.306	-0.09	-0.27 - 0.09
**Random Part**					
σ^2	0.238				
$\tau_{00, ID}$	0.428				
ρ_{01}	-0.280				
N_{ID}	50				
ICC_{ID}	0.643				
Observations	1000				
R^2	0.864				

Note: touch type (CT-targeted, control), multiplication factor (3, 6). Random slopes: multiplication factor x touch type.

Table 3. *Linear mixed model results of return ratio in experiment 2 predicted by multiplication factor, touch type, touch pleasantness rating and their interactions.*

	Return Ratio - experiment 2				
	χ^2	df	p	std. Beta	95% CI
**Fixed Part**					
Multiplication Factor	131.020	1	<0.001	-0.50	-0.66 - -0.34
Touch Type	0.442	1	0.506	0.13	-0.07 - 0.34
Pleasantness Rating	4.602	1	0.032	0.02	-0.05 - 0.10
Multiplication Factor x Touch Type	0.097	1	0.756	-0.02	-0.16 - 0.12
Multiplication Factor x Pleasantness Rating	0.734	1	0.392	0.06	-0.08 - 0.21
Touch Type x Pleasantness Rating	2.411	1	0.121	-0.16	-0.36 - 0.04
Multiplication Factor x Touch Type x Pleasantness Rating	0.200	1	0.655	0.03	-0.11 - 0.18
**Random Part**					
σ^2	0.211				
$\tau_{00, ID}$	0.517				
ρ_{01}	-0.959				
N_{ID}	79				
ICC_{ID}	0.710				
Observations	1580				
R^2	0.837				

Note: touch type (CT-targeted, control), multiplication factor (3, 6). Random slopes: multiplication factor x touch type.

Results 2

Table 4. *Linear mixed model results of combined data from experiment 1 and 2, of return ratio predicted by multiplication factor, touch type, touch pleasantness rating, hypothesis guessing and their interactions.*

	Return Ratio – experiment 1 and 2 pooled together				
	χ^2	df	p	std. Beta	95% CI
**Fixed Part**					
Multiplication Factor (MF)	347.913	1	<0.001	-0.34	-0.45 - -0.23
Touch Type (TT)	0.173	1	0.678	0.00	-0.16 - 0.17
Pleasantness rating (PR)	0.432	1	0.511	0.03	-0.04 - 0.10
Hypothesis guessed (HG)	0.368	1	0.544	-0.13	-0.29 - 0.03
MF x TT	1.329	1	0.249	0.06	-0.04 - 0.17
MF x PR	0.028	1	0.868	-0.01	-0.17 - 0.15
TT x PR	0.011	1	0.915	-0.01	-0.20 - 0.18
MF x HG	15.788	1	<0.001	0.22	0.11 - 0.33
TT x HG	0.013	1	0.910	-0.01	-0.18 - 0.16
PR x HG	2.038	1	0.153	0.12	-0.04 - 0.28
MF x TT x PR	1.314	1	0.252	-0.08	-0.22 - 0.06
MF x TT x HG	0.139	1	0.709	-0.02	-0.13 - 0.09
MF x PR x HG	7.226	1	0.007	-0.22	-0.38 - -0.06
TT x PR x HG	0.059	1	0.808	-0.02	-0.21 - 0.17
MF x TT x PR x HG	0.387	1	0.534	0.04	-0.10 - 0.18
**Random Part**					
σ^2	0.227				
$\tau_{00, \text{experiment:ID}}$	0.365				
ρ_{01}	-0.475				
$N_{\text{experiment:ID}}$	129				
$ICC_{\text{experiment:ID}}$	0.616				
Observations	2580				
R^2	0.850				

Note: touch type (CT-targeted, control), multiplication factor (3, 6), hypothesis guessed (yes, no). Random slopes: pleasantness rating x multiplication factor x touch type.

Table 5. *Results of post-hoc linear mixed model analysis of significant multiplication factor x pleasantness rating x hypothesis guessing interaction. Results of linear mixed model for participants who correctly guessed hypothesis.*

	Return Ratio - Hypothesis guessed correctly				
	χ^2	df	p	std.Beta	95% CI
**Fixed Part**					
Multiplication Factor (MF)	11.992	1	< 0.001	-0.08	-0.26 - 0.10
Touch Type (TT)	1.622	1	0.203	0.03	-0.21 - 0.27
Pleasantness rating (PR)	0.451	1	0.502	0.12	-0.03 - 0.28
MF x TT	0.207	1	0.649	0.04	-0.12 - 0.20
MF x PR	3.999	1	0.046	-0.26	-0.52 - -0.01
TT x PR	0.381	1	.0537	-0.07	-0.30 - 0.16
MF x TT x PR	0.120	1	0.729	-0.03	-0.20 - 0.14
**Random Part**					
σ^2	0.243				
$\tau_{00,\ experiment:ID}$	0.505				
ρ_{01}	-0.784				
$N_{experiment:ID}$	20				
$ICC_{experiment:ID}$	0.675				
Observations	400				
R^2	0.834				

Note: touch type (CT-targeted, control), multiplication factor (3, 6). Random slopes: pleasantness rating x multiplication factor.

Table 6. *Results of post-hoc linear mixed model analysis of significant multiplication factor x pleasantness rating x hypothesis guessing interaction. Results of linear mixed model for participants who did not guess hypothesis.*

	Return Ratio - Hypothesis not guessed correctly				
	χ^2	df	p	std. Beta	95% CI
**Fixed Part**					
Multiplication Factor (MF)	357.473	1	< 0.001	-0.56	-0.64 - -0.48
Touch Type (TT)	0.804	1	0.370	0.02	-0.10 - 0.14
Pleasantness rating (PR)	0.255	1	0.614	-0.03	-0.08 - 0.03
MF x TT	3.913	1	0.048	0.08	0.00 - 0.16
MF x PR	11.148	1	< 0.001	0.20	0.08 - 0.32
TT x PR	0.013	1	0.908	0.01	-0.13 - 0.15
MF x TT x PR	4.912	1	0.027	-0.12	-0.23 - -0.01
**Random Part**					
σ^2	0.223				
$\tau_{00,\ experiment:ID}$	0.413				
ρ_{01}	-0.544				
$N_{experiment:ID}$	109				
$ICC_{experiment:ID}$	0.649				

Observations	2180
R^2	0.852

Note: touch type (CT-targeted, control), multiplication factor (3, 6). Random slopes: pleasantness rating x multiplication factor x touch type.

Results 3

Table 7. *Post-hoc comparison of significant multiplication factor x touch type x pleasantness rating interaction of linear mixed model displayed in Table 6. Results are from combined data from experiment 1 and 2 of participants who did not guess the study´s hypothesis and show return ratios predicted by multiplication factor, touch pleasantness rating and their interaction for the CT-targeted touch condition.*

	Return Ratio - hypothesis not guessed CT-targeted touch				
	χ^2	df	p	std. Beta	95% CI
**Fixed Part**					
Multiplication factor	236.976	1	< 0.001	-0.53	-0.65 - -0.41
Pleasantness rating	0.007	1	0.934	-0.06	-0.17 - 0.04
Multiplication factor x Pleasantness rating	2.685	1	0.101	0.12	-0.02 - 0.27
**Random Part**					
σ^2	0.177				
$\tau_{00,\ experiment:ID}$	0.255				
ρ_{01}	-0.231				
$N_{experiment:ID}$	109				
$ICC_{experiment:ID}$	0.590				
Observations	1090				
R^2	0.874				

Note: multiplication factor (3, 6). Random slopes: pleasantness rating x multiplication factor.

Table 8. *Post-hoc comparison of significant multiplication factor x touch type x pleasantness rating interaction of linear mixed model displayed in Table 6. Results are from combined data from experiment 1 and 2 of participants who did not guess the study´s hypothesis and show return ratios predicted by multiplication factor, touch pleasantness rating and their interaction for the control touch condition.*

	Return Ratio - hypothesis not guessed control touch				
	χ^2	df	p	std. Beta	95% CI
**Fixed Part**					
Multiplication factor	176.559	1	< 0.001	-0.67	-0.80 - -0.54
Pleasantness rating	1.241	1	0.265	-0.11	-0.24 - 0.01
Multiplication factor x Pleasantness rating	13.234	1	< 0.001	0.34	0.16 - 0.52
**Random Part**					
σ^2	0.274				
$\tau_{00,\ experiment:ID}$	0.186				
ρ_{01}	0.575				

$N_{experiment:ID}$	109
$ICC_{experiment:ID}$	0.403
Observations	1090
R^2	0.836

Note: multiplication factor (3, 6). Random slopes: pleasantness rating x multiplication factor.

Results 4

Table 9. *Linear mixed model results from data from experiment 3 of SVO angle predicted by stimulation type, touch pleasantness rating and their interaction.*

	SVO angle				
	χ^2	df	p	std. Beta	95% CI
**Fixed Part**					
Stimulation type	5.756	2	0.056		
Control stimulation				0.14	-0.26 - 0.53
CT-targeted stimulation				-0.25	-0.65 - 0.15
visual stimulation				0.11	-0.38 - 0.60
Pleasantness rating	1.164	1	0.281	-0.08	-0.21 - 0.04
Stimulation type x Pleasantness Rating	2.824	2	0.244		
Control stimulation x pleasantness rating				-0.26	-0.66 - 0.14
CT-targeted stimulation x pleasantness rating				0.28	-0.12 - 0.69
visual stimulation x pleasantness rating				0.01	-0.51 - 0.52
**Random Part**					
σ^2	25.717				
$\tau_{00, ID}$	190.703				
ρ_{01}	-0.885				
N_{ID}	44				
ICC_{ID}	0.881				
Observations	128				
R^2	0.867				

Note: stimulation type (CT-targeted, control, visual). Random slope: pleasantness rating.
